# Molecular Features of Triple Negative Breast Cancer: Microarray Evidence and Further Integrated Analysis

**DOI:** 10.1371/journal.pone.0129842

**Published:** 2015-06-23

**Authors:** Jinsong He, Jianbo Yang, Weicai Chen, Huisheng Wu, Zishan Yuan, Kun Wang, Guojin Li, Jie Sun, Limin Yu

**Affiliations:** 1 Department of Breast Surgery, The first affiliated hospital of Shenzhen university, the Second People’s Hospital of Shenzhen, Shenzhen 518035, China; 2 Department of Laboratory Medicine and Pathology, Masonic Cancer Center, University of Minnesota, UMN Twin Cities, Minneapolis 55455, Minnesota, United States of America; Cleveland Clinic Lerner Research Institute, UNITED STATES

## Abstract

**Purpose:**

Breast cancer is a heterogeneous disease usually including four molecular subtypes such as luminal A, luminal B, HER2-enriched, and triple-negative breast cancer (TNBC). TNBC is more aggressive than other breast cancer subtypes. Despite major advances in ER-positive or HER2-amplified breast cancer, there is no targeted agent currently available for TNBC, so it is urgent to identify new potential therapeutic targets for TNBC.

**Methods:**

We first used microarray analysis to compare gene expression profiling between TNBC and non-TNBC. Furthermore an integrated analysis was conducted based on our own and published data, leading to more robust, reproducible and accurate predictions. Additionally, we performed qRT-PCR in breast cancer cell lines to verify the findings in integrated analysis.

**Results:**

After searching Gene Expression Omnibus database (GEO), two microarray studies were obtained according to the inclusion criteria. The integrated analysis was conducted, including 30 samples of TNBC and 77 samples of non-TNBC. 556 genes were found to be consistently differentially expressed (344 up-regulated genes and 212 down-regulated genes in TNBC). Functional annotation for these differentially expressed genes (DEGs) showed that the most significantly enriched Gene Ontology (GO) term for molecular functions was protein binding (GO: 0005515, P = 6.09E-21), while that for biological processes was signal transduction (GO: 0007165, P = 9.46E-08), and that for cellular component was cytoplasm (GO: 0005737, P = 2.09E-21). The most significant pathway was Pathways in cancer (P = 6.54E-05) based on Kyoto Encyclopedia of Genes and Genomes (KEGG). DUSP1 (Degree = 21), MYEOV2 (Degree = 15) and UQCRQ (Degree = 14) were identified as the significant hub proteins in the protein-protein interaction (PPI) network. Five genes were selected to perform qRT-PCR in seven breast cancer cell lines, and qRT-PCR results showed that the expression pattern of selected genes in TNBC lines and non-TNBC lines was nearly consistent with that in the integrated analysis.

**Conclusion:**

This study may help to understand the pathogenesis of different breast cancer subtypes, contributing to the successful identification of therapeutic targets for TNBC.

## Introduction

Breast cancer is a heterogeneous disease usually composed of four molecular subtypes including luminal A, luminal B, HER2-enriched, and triple-negative breast cancer (TNBC) [1]. TNBC is defined by negative of expression of the ER, PR, and HER2 amplification, accounting for approximately 15% of all breast cancers. Despite major advances in ER-positive or HER2-amplified breast cancers, there is no targeted agent currently available for TNBC, leaving cytotoxic chemotherapy as the only option for systemic therapy[2]. In addition, TNBC is more aggressive than other breast cancer subtypes for its propensity for recurrence and metastasis, causing that the prognosis for TNBC patients is very poor [3]. Therefore, it is urgent to identify new potential therapeutic targets for TNBC.

The high-throughput technologies allow simultaneous examination of the global gene expression, and have been used in many fields. The application of these technologies could categorize the characteristics of different subtypes of cancers, and identify genes that may be used as novel molecular targets for therapeutic modalities[4]. Gene expression profiling has stratified breast cancer into discrete biologic subtypes that largely associated with the expression status of ER, PR, and Her2 in tumor cells[5], contributing to the molecular biology of the disease in a subtype specific manner. Xi Chen et al. discovered six TNBC subtypes from 587 TNBC samples based on gene expression patterns, developing a subtyping tool for TNBC[6]. Komatsu et al. performed microarray analysis on 30 TNBC and 13 normal epithelial ductal cells, identified differentially expressed genes (DEGs) involved in cell cycle such as ASPM and CENPK which mediated the cell viability of TNBC[7]. Recently a integrated analysis has been conducted in the Oncomine database to identify 206 deregulated genes [8] in TNBC compared with non-TNBC and these genes was also found to be deregulated in tumors that metastasized or led to death within 5 years, enriching in two core biological functions: CIN and ER signaling. In this integrated analysis the heterogeneity was increased due to clinical samples experiencing different chemotherapy among multiple datasets. However in our integrated analysis, we first used microarray analysis to identify differentially expressed genes (DEGs) and biological processes associated with TNBC. Furthermore we aim to back up our result by conducting a integrated analysis of our own and published gene expression data of TNBC in breast tissues without drug treatment, leading to more robust, reproducible and accurate predictions[9]. To verify the findings in the integrated analysis, some genes were selected to perform qRT-PCR in breast cancer cell lines. Our study adds a novel insight into the understanding of pathological mechanism underlying breast cancers. In addition, it would help to identify putative therapeutic targets in treating different subtypes of breast cancer.

## Materials and Methods

### Patients and tissues

The breast tissues of different breast cancer subtypes (including 2 samples of TNBC, 1 sample of LuminalA, 1 sample of LuminalB and 4 samples of HER2+) were provided by the Second People’s Hospital of Shenzhen, with the approval of patients and hospital authorities. All protocols were approved by the Second People’s Hospital of Shenzhen Medical Ethics Committee. The written informed consent forms were obtained from patients or legal guardians of the patients. Clinical information of the patients was abstracted from medical records and they were diagnosed as triple-negative by pathologists with immunohistochemical staining showing ER-negative, PR-negative, and HER2 (0 or 1+). The tissues were obtained from biopsies and stored in 1ml RNAlater solution.

### Microarray analysis

RNA extracted using the Qiagen RNeasy kit according to the manufacturer instructions. Human HT expression beadchip V4 array (illumina) was performed to detect gene expression profiles of breast cancer samples. GenomeStudio (Illumina) was used to process and analyze raw signal intensities of gene expression data, with background correction, quantile normalization conducted on the average signal intensities. P-value was computed. Fold changes in gene expression, which is the ratio of average expression level between TNBC and non-TNBC group, were calculated. The Illumina customer model employed multiple testing corrections to compute the false discovery rate (FDR). Differentially expressed genes (DEGs) between TNBC and non-TNBC samples were subsequently determined. Heat map analysis was conducted using the “heatmap.2” function of the R/Bioconductor package “gplots”[10].

### Integrated analysis of microarray datasets

To obtain maximal information regarding the difference of gene expression profiling between TNBC and non-TNBC in the present study, integrated analysis was performed combining the microarray data presented here together with all previous microarray data. To this end, we searched the Gene Expression Omnibus database (GEO, http://www.ncbi.nlm.nih.gov/geo) [11] with keywords “triple-negative breast cancer, gene expression, microarray, genetics”. We only selected the original experimental microarray studies that analyzed gene expression profiling of breast tumor tissues between TNBC and non-TNBC in human. Expression profiles obtained by integrated analysis or with other treatment were excluded.

Different gene IDs or probe IDs was uniformly converted to Entrez IDs. Intensity values of each probe-set were log2 transformed, and Z-score were calculated to incorporate between-study heterogeneities across multiple studies [12]. A gene specific t-test was carried out between TNBC and non-TNBC, then p-value and effect size of each microarray study were calculated. P-value from multiple studies was combined using Fisher test, and the random effects model was used to combine effect size from multiple studies. We selected genes with combined p-value < 0.01 and the absolute value of combined ES > 1 as DEGs.

### Functional annotation of DEGs

Gene Ontology (GO) enrichment analysis was carried out to uncover the biological functions of the DEGs. Additionally, the biological pathway enrichment analysis of all DEGs was performed based on the Kyoto Encyclopedia of Genes and Genomes (KEGG) database. The online based software GENECODIS, a function analysis tool, which integrated kinds of information resources (GO, KEGG or SwissProt) was available to perform these analyses [13].

### PPI Network Construction

The protein-protein interactions (PPIs) analysis could visualize functional links between DEGs and other genes at the molecular level[14], which is useful to help understand the molecular mechanism of diseases and its potentially essential genes. In our present study, PPI network of the top 10 up- and down-regulated DEGs was established based on Biological General Repository for Interaction Datasets (BioGRID) (http://thebiogrid.org/), and visualized with Cytoscape software[15] to find the hub proteins of the network.

### QRT-PCR validation

The total RNA from each sample was extracted using Trizol method (BeijingDingGuoChangSheng Biotechnology Co., Ltd.). Primer 5.0 software (PREMIER Biosoft, Palo Alto, CA) was utilized to design primers for SYBR-Green experiments based on template sequences, and an ABI 7500 Real Time PCR System (Applied Biosystems, Carlsbad CA) was used. For each replicate, cDNA was synthesized from 1–5 μg RNA using Superscript Reverse Transcriptase II (TOYOBO). The qRT-PCR reaction consisted of 12.5 μl of Power SYBR Green PCR Master Mix (Applied Biosystems/Life Technologies, Carlsbad, CA), 1 μl diluted cDNA and 0.25 μl of each primer (10μM)) contributing a total volume of 25 μl. Reactions were conducted in triplicate, and run in 96-well plates as the following conditions: 94°C for 2 min, 35 cycles of 94°C for 30 sec, 55°C for 30 sec, 72°C for 30 sec, and 72°C for 10 min. Relative gene expression was analyzed using Data Assist Software version 3.0 (Applied Biosystems/Life Technologies), using human GAPDH gene as endogenous controls for RNA load and gene expression in analysis.

## Result

### Microarray analysis

**Fig 1:**
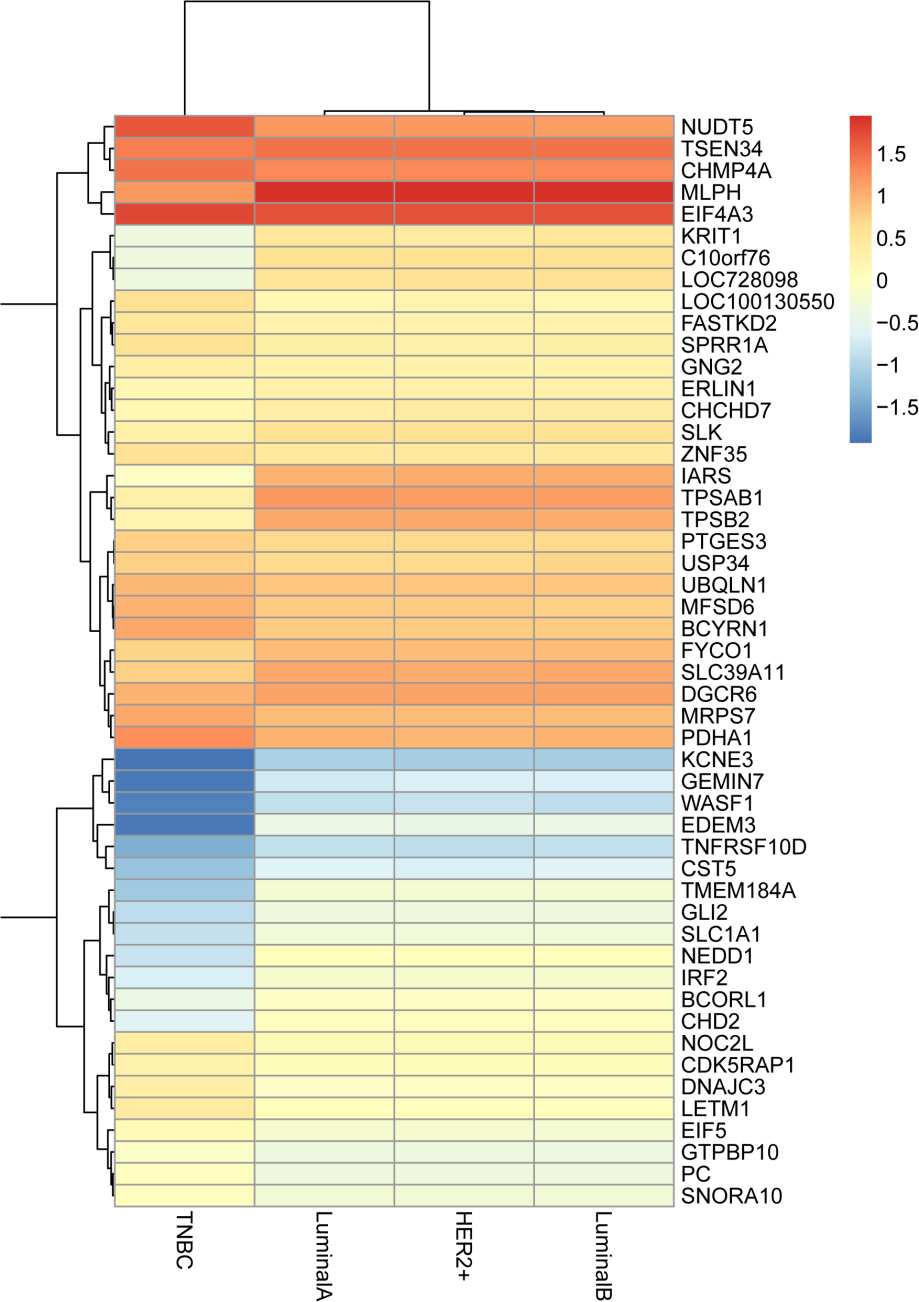
Heat-map image representing 66 genes that were significantly up-regulated or down-regulated (p<0.003) in TNBC compared with other breast cancer subtypes including LuminalA, LuminalB and HER2+.

We selected 8 cases of breast cancers including 2 cases of TNBC and 6 cases of non-TNBC, which were identified by immuno-histochemical staining of ER, PR and HER2. The clinical and pathological features of the samples are summarized in Table 1. To delineate the changes in gene expression patterns between TNBC and non-TNBC, we performed microarray analysis. The DEGs with p < 0.003 were identified and displayed in a heat map across different subtypes of breast cancers (Fig 1).

**Table 1:** Clinical characteristics of breast cancer patients for microarray analysis.

Molecular Subtype	Patient ID	Age	Tumor size(cm)	Histological type	TNM	Stage
TNBC	4	47	2.2cm×1.7cm×1.3cm	IDC	T2N3M0	Ⅲ
	8	41	3.5cm×3cm×1.8cm	IDC	T2N2M0	Ⅲ
LuminalB	5	45	1.5cm×1cm×1cm	IDC	T1cN1M0	Ⅰ∼Ⅱ
LuminalA	9	42	1.3cm×0.9cm×0.6cm	DCIS	T1N0M0	I
HER2+	6	53	4cm×2.5cm×1.5cm	DCIS	T2N0M0	Ⅱ
	7	44	2cm×1.5cm×1.5cm	IDC	T1N1M0	Ⅱ
	11	60	3.5cm×2.5cm×2cm	IDC	T2N2M0	Ⅱ
	12	61	2.3cm×2cm×2cm	IDC	T2N0M0	Ⅲ

IDC: infiltrating ductal carcinoma, DCIS: ductal carcinoma in situ.

### DEGs in the integrated analysis of microarray datasets

After electronic search, only two microarray studies were obtained according to the inclusion criteria (S1 Table). We subsequently combined these microarray studies to our own data to perform an integrated analysis which it contained 30 samples of TNBC and 77 samples of non-TNBC. The individual studies for integrated analysis were displayed in Table 2. The integrated analysis showed that a total of 556 genes were found to be consistently differentially expressed with 344 genes up-regulated and 212 genes down-regulated in TNBC (S2 Table). The top 10 most significantly up- or down-regulated genes were listed in Table 3. COL4A2 (collagen, type IV, alpha 2), the most significantly up-regulated gene (p-value = 1.46E-10), encodes one of major structural components of basement membranes. Previous studies found that COL4A2 was significantly overexpressed in ER-positive breast cancer compared to ER-negative breast cancer[16]. CMBL (carboxymethylenebutenolidase homolog (Pseudomonas)), as a cysteine hydrolase of the dienelactone hydrolase family, was found to be the most significant down-regulated gene (p = 4.29E-09).

**Table 2:** Characteristics of the individual studies for integrated analysis.

GEO ID	Platform	Sample count (TNBC:other BC)	Author	Time	Country
GSE27447	GPL6244 [HuGene-1_0-st] Affymetrix Human Gene 1.0 ST Array	5:14	Yang L	2011	USA
GSE18864	GPL570 [HG-U133_Plus_2] Affymetrix Human Genome U133 Plus 2.0	24:60	Silver DP	2010	Denmark
Local data	Human HT expression beadchip V4	1:3	He J	2014	China

**Table 3:** Top 15 most significantly up- or down-regulated DEGs.

Gene ID	Gene Symbol	combined ES	combined p-value (FDR)
**Up-regulated genes**			
1284	COL4A2	-2.0761	1.46E-10
55122	AKIRIN2	-1.9121	2.42E-09
29886	SNX8	-1.8678	4.02E-09
51621	KLF13	-1.8756	5.22E-09
79786	KLHL36	-1.7548	1.67E-08
9909	DENND4B	-1.7897	2.28E-08
283149	BCL9L	-1.8704	2.55E-08
23765	IL17RA	-1.7008	6.07E-08
90427	BMF	-1.7394	6.11E-08
9258	MFHAS1	-1.7234	6.46E-08
221061	FAM171A1	-1.7851	7.27E-08
92241	RCSD1	-1.6946	8.62E-08
7187	TRAF3	-1.6906	8.66E-08
55689	YEATS2	-1.6551	1.07E-07
7841	MOGS	-1.6698	1.45E-07
**Down-regulated genes**			
134147	CMBL	1.9233	4.29E-09
1843	DUSP1	1.7381	2.17E-08
153562	MARVELD2	1.7058	8.99E-08
26996	GPR160	1.8247	1.06E-07
3169	FOXA1	2.3038	1.67E-07
57669	EPB41L5	1.6701	2.26E-07
158158	RASEF	1.6221	2.62E-07
27089	UQCRQ	1.5901	3.71E-07
150678	MYEOV2	1.6048	4.06E-07
79083	MLPH	11.993	5.48E-07
79875	THSD4	2.1243	8.41E-07
150590	C2orf15	1.5304	8.98E-07
10551	AGR2	1.6211	1.25E-06
91074	ANKRD30A	1.5391	1.30E-06
79858	NEK11	1.4965	1.35E-06

### Functional annotation

**Fig 2:**
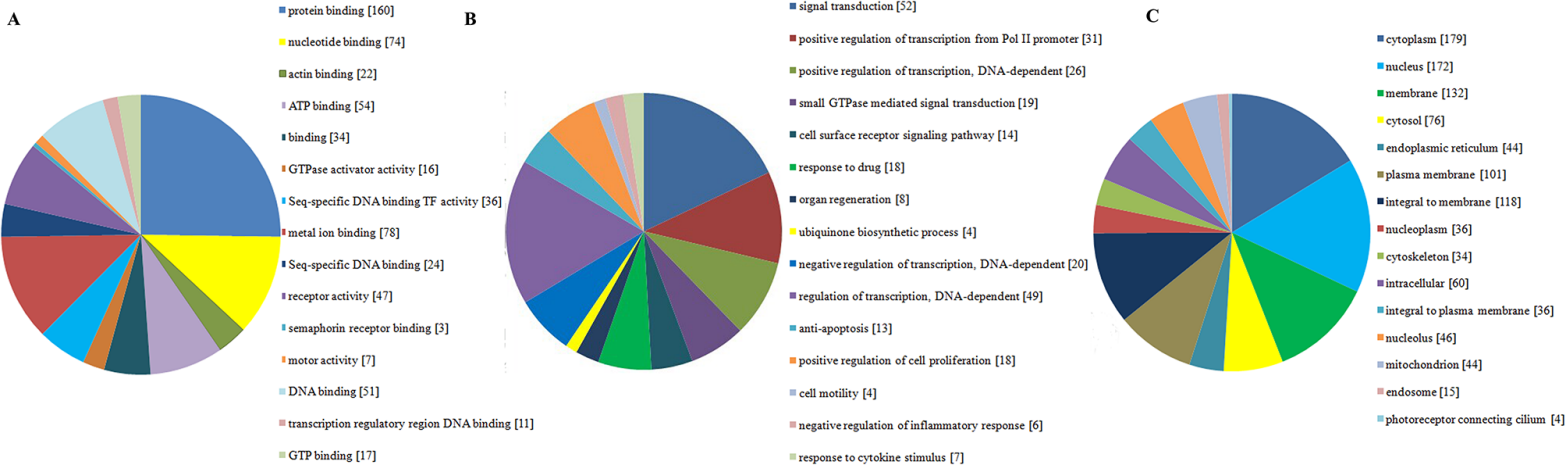
The top 15 enriched GO terms of differentially expressed genes. A. molecular functions for DEGs (p value ≤ 9.35E-06); B. biological process for DEGs (p value ≤ 1.92E-07); C. cellular component for DEGs (p value ≤ 2.98E-09).

GO enrichment analysis of DEGs was performed to understand their biological functions. GO categories are separated into 3groups: molecular function, biological process and cellular component. In our analysis, the 3 categories were detected respectively using web-based software GENECODIS. We found that the significantly enriched GO terms for molecular functions were protein binding (GO: 0005515, p = 6.09E-21) and nucleotide binding (GO: 0000166, p = 7.47E-08), while those for biological processes were signal transduction (GO:0007165, p = 9.46E-08) and positive regulation of transcription from RNA polymerase II promoter (GO: 0045944, p = 5.41E-06) and those for cellular component were cytoplasm (GO: 0005737, p = 2.09E-21) and nucleus (GO: 0005634, p = 1.38E-17)(Fig 2).

The pathway enrichment analysis was also conducted to further evaluate the biological pathways the DEGs involved. Hypergeometric test was carried out with p-value < 0.05 as the criteria for significant pathway identification (Table 4). The most significantly enriched pathway was Pathways in cancer (p = 6.54E-05). Furthermore, Jak-STAT signaling pathway (p = 4.35E-04) and Osteoclast differentiation (p = 5.62E-04) were found to be significantly enriched.

**Table 4:** Top 15 enriched KEGG pathway of DEGs.

KEGG ID	KEGG term	No. of genes	*F*.*D*.*R*
hsa05200	Pathways in cancer	20	6.54E-05
hsa04630	Jak-STAT signaling pathway	12	4.35E-04
hsa04380	Osteoclast differentiation	11	5.62E-04
hsa04670	Leukocyte transendothelial migration	10	6.55E-04
hsa04650	Natural killer cell mediated cytotoxicity	10	7.88E-04
hsa04142	Lysosome	10	7.93E-04
hsa04664	Fc epsilon RI signaling pathway	8	8.43E-04
hsa05130	Pathogenic Escherichia coli infection	7	8.62E-04
hsa05222	Small cell lung cancer	8	1.23E-03
hsa05215	Prostate cancer	8	1.54E-03
hsa00130	Ubiquinone and other terpenoid-quinone biosynthesis	3	2.21E-03
hsa05162	Measles	9	2.72E-03
hsa04662	B cell receptor signaling pathway	7	2.78E-03
hsa04070	Phosphatidylinositol signaling system	7	2.80E-03
hsa04974	Protein digestion and absorption	7	2.88E-03

### PPI network construction

**Fig 3:**
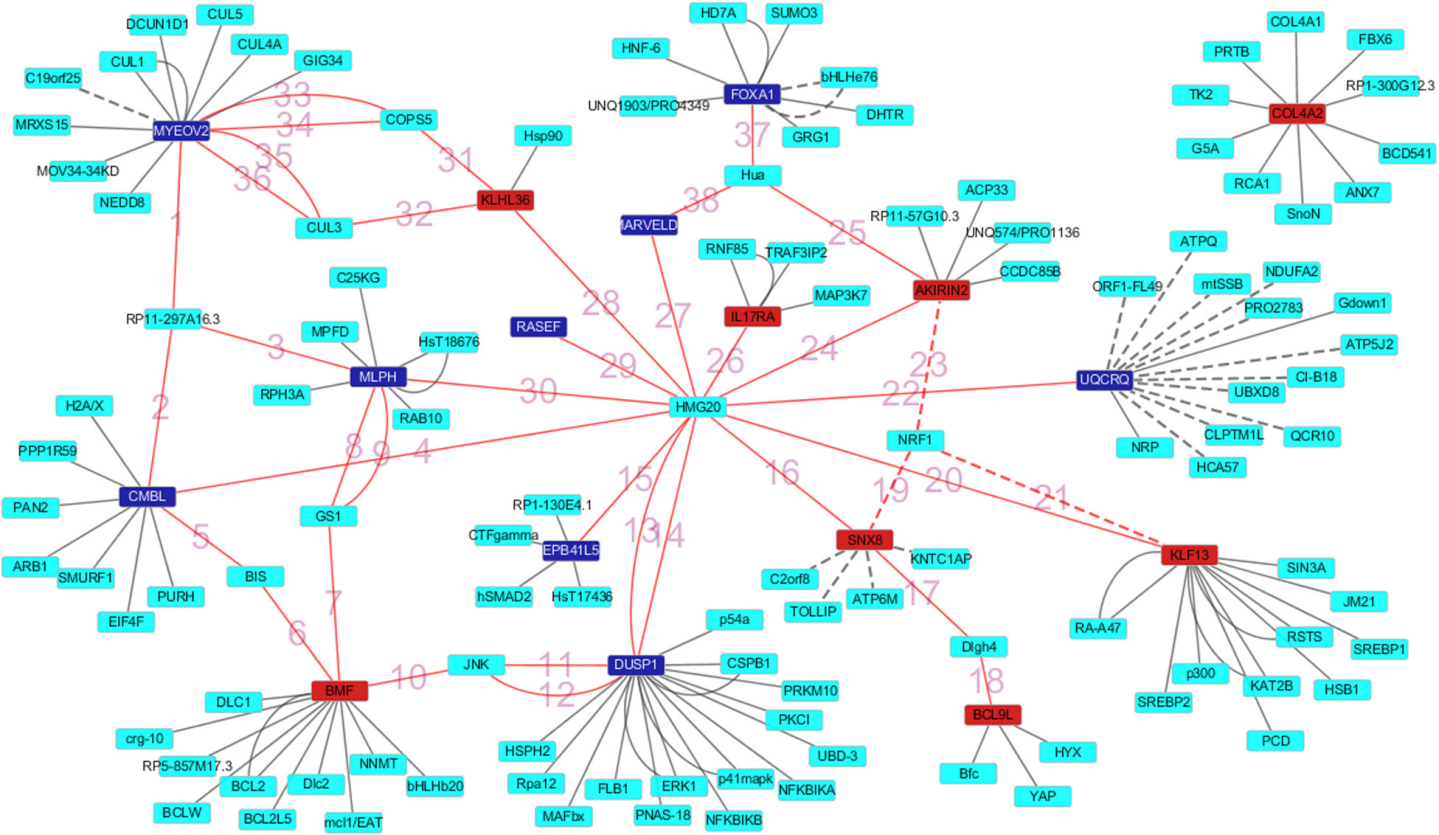
The constructed PPI network of the top 10 up- and down-regulated DEGs. Nodes denote proteins, edges denote interactions between two proteins.

The PPI networks of the top 20 most significantly dysregulated genes were established including 221 nodes, 160 edges. The significant hub proteins contained DUSP1 (dual specificity phosphatase 1, Degree = 21), MYEOV2 (myeloma overexpressed 2, Degree = 15) and UQCRQ (ubiquinol-cytochrome c reductase, complex III subunit VII, 9.5kDa, Degree = 14) (Fig 3). In addition we labeled the edges connecting top 20 most significantly dysregulated genes directly or indirectly with numbers in Fig 3 and provided annotation in S3 Table.

### QRT-PCR validation

To validate the findings in the integrated analysis, we selected 5 genes (COL4A2, BMF, DUSP1, FOXA1, MLPH) of the top 10 up or down regulated genes to perform qRT-PCR in seven human breast cancer cell lines including three TNBC lines (MDA-MB-231, MDA-MB-435, MDA-MB-468) and four non-TNBC lines (MDA-MB-453, MCF-7, BT-474, SK-BR-3). COL4A2 and BMF were selected as the up-regulated genes in TNBC, while DUSP1, FOXA1, and MLPH were selected as the down-regulated genes in TNBC.

**Fig 4:**
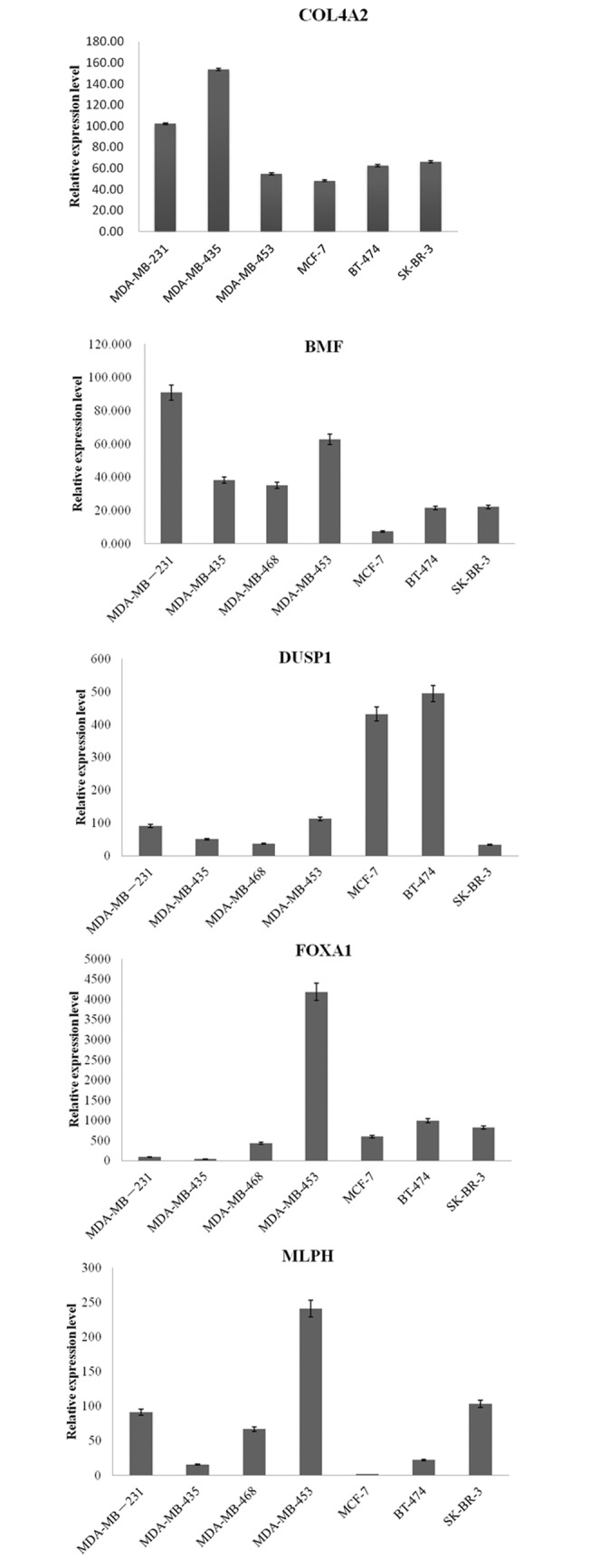
qRT-PCR validation of five genes (COL4A2, BMF, DUSP1, FOXA1and MLPH) in seven breast cancer cell lines (MDA-MB-231, MDA-MB-435, MDA-MB-468, MDA-MB-453, MCF-7, BT-474 and SK-BR-3).

The qRT-PCR results showed that the expression pattern of selected genes in TNBC lines and non-TNBC lines were nearly consistent with that in the integrated analysis. As displayed in Fig 4, the expression of COL4A2 was dramatically higher in MDA-MB-231, MDA-MB-435 which were both TNBC lines than that in non-TNBC lines including MDA-MB-453, MCF-7, BT-474, SK-BR-3. TNBC lines expressed higher level of BMF when compared with non-TNBC lines of MCF-7, BT-474, SK-BR-3, while MDA-MB-453, as a HER2-positive breast cancer cell line, also expressed high level of BMF. For DUSP1 and FOXA1 genes, their expression in TNBC lines is lower than that in non-TNBC lines, while this trend was not obvious for MLPH, with lower expression in TNBC lines when compared two of non-TNBC lines including MDA-MB-453 and SK-BR-3. In addition, we found that DUSP1 was significantly over-expressed in ER-positive breast cancer cell lines (MCF-7, BT-474) as compared to ER-negative breast cancer cell lines (MDA-MB-231, MDA-MB-435, MDA-MB-468, MDA-MB-453, SK-BR-3).

## Discussion

The molecular signatures of breast cancer subtypes not only reflect the biological features of the corresponding malignant neoplasms, but also predict their clinical behavior and responses to given therapy, with some subtypes having better outcomes than others[17, 18]. In this paper, gene expression analysis was performed by microarray to compare gene expression profiling of TNBC to that of non-TNBC, followed by integrated analysis of our own and published expression data to identify key genes and biological pathways directing the development of effective targeted therapies for TNBC. In total, 556 genes (344 genes up-regulated and 212 genes down-regulated in TNBC) were consistently expressed differentially between TNBC and non-TNBC in the integrated analysis. COL4A2 was the most significant up-regulated gene in this integrated analysis, and this high expression in TNBC was further confirmed by qRT-PCR. COL4A2 was also found to be significantly overexpressed in ER-negative breast cancer compared with ER-positive breast cancer[16], which was in line with our finding. Among the top 10 up-regulated DEGs, BMF (BCL2-modifying factor), which interacts with prosurvival Bcl-2 family members to trigger apoptosis[19], was down-regulated in MCF-7 breast cancer cells as a consequence of induced NeuT overexpression[20]. In qRT-PCR result, the expression of BMF in MCF-7 breast cancer cells was also lower than the other breast cancer cell lines. The role of BMF on the development of TNBC was unknown.

CMBL (carboxymethylenebutenolidase homolog (Pseudomonas)), the most significant down-regulated gene, was found to be highly expressed in liver cytosol, but the association with breast cancer wasn’t reported. In our study, several genes of the top 10 down-regulated DEGs such as DUSP1, FOXA1, MLPH was implicated in the development of breast cancer. DUSP1 (also known as MKP-1), a member of the dual-specificity phosphatases (DUSPs) which interacted and catalyzed dephosphorylation of active MAPK, mediated anti-proliferative and anti-inflammatory actions of PR in the breast cancer[21]. In this study, we found that the expression of DUSP1 was lower in TNBC than that in non-TNBC by integrated analysis and qRT-PCR, and may be associated with ER status. Furthermore our results of PPI network also detected the important role of DUSP1, which may be considered as a potential target gene for the treatment of TNBC. As a key determinant of estrogen receptor function and endocrine response[22], FOXA1 participated with ERα and GATA3 in a complex transcriptional regulatory program to drive tumor growth[23] in estrogen receptor-α (ERα)-positive breast tumors, so the absence of estrogen receptor may lead to the decrease of FOXA1 expression. Our result indicated that FOXA1 expression was lower in TNBC as compared to non-TNBC. MLPH was found to be significantly over-expressed in ERα (+) breast tumors as compared to ERα (-) breast tumors[24] by microarray analysis and RT-qPCR confirmation, while this was not observed in our study, and the main reason may be utilization of different sample source and sample size.

Pathways in cancer and Jak-STAT signaling pathway were found to be highly enriched for the DEGs. Amounts of pathways were implicated in the development of breast cancer. Importantly, we noted that the most significantly enriched pathway was Pathways in cancer, which refers to some classical pathways such as Wnt signaling pathway, PI3K-Akt signaling pathway, Jak-STAT signaling pathway, MAPK signaling pathway, ErbB signaling pathway and some others together to regulate tumor growth and metastasis[25]. This finding further confirms that TNBC is more aggressive than other breast cancer subtype. Poage GM et al. suggested that the JAK/STAT was one of TNBC-specific regulatory feedback programs, and its critical signaling nodes could be used as the therapy target for the treatment of TNBC[26].

In conclusion, by the integrated analysis and qRT-PCR validation, we showed the underlying molecular differences between TNBC and other breast cancer subtypes, and identified DEGs and biological function, which may help to understand the pathogenesis of different breast cancer subtypes and the development of drug therapy for TNBC. Further experiments in vivo and vitro are needed to uncover the biological function of the differentially regulated genes in the pathogenesis of TNBC.

## Supporting Information

S1 Table The completed PRISMA checklist. (DOCX)

S2 Table The full list of DEGs between TNBC and non-TNBC in the integrated-analysis. (DOC)

S3 Table The annotation of edges connecting the top 10 up- and down-regulated DEGs directly or indirectly. (DOC)
