# Supplementary material for: Molecular Features of Triple Negative Breast Cancer: Microarray Evidence and Further Integrated Analysis
Source: PLoS One. 2015 Jun 23;10(6):e0129842. doi: 10.1371/journal.pone.0129842 (PMC4478040; doi:10.1371/journal.pone.0129842)
Supplement: S2 Table — (DOC) [file pone.0129842.s002.doc]

**S2 Table. The full list of DEGs between TNBC and non-TNBC in the integrated-analysis**

1. Up-regulated genes

| **Gene ID** | **Gene Symbol** | **combined ES** | **combined p-value(fdr)** |
| --- | --- | --- | --- |
| 1284 | COL4A2 | -2.0761 | 1.46E-10 |
| 55122 | AKIRIN2 | -1.9121 | 2.42E-09 |
| 29886 | SNX8 | -1.8678 | 4.02E-09 |
| 51621 | KLF13 | -1.8756 | 5.22E-09 |
| 79786 | KLHL36 | -1.7548 | 1.67E-08 |
| 9909 | DENND4B | -1.7897 | 2.28E-08 |
| 283149 | BCL9L | -1.8704 | 2.55E-08 |
| 23765 | IL17RA | -1.7008 | 6.07E-08 |
| 90427 | BMF | -1.7394 | 6.11E-08 |
| 9258 | MFHAS1 | -1.7234 | 6.46E-08 |
| 221061 | FAM171A1 | -1.7851 | 7.27E-08 |
| 92241 | RCSD1 | -1.6946 | 8.62E-08 |
| 7187 | TRAF3 | -1.6906 | 8.66E-08 |
| 55689 | YEATS2 | -1.6551 | 1.07E-07 |
| 7841 | MOGS | -1.6698 | 1.45E-07 |
| 23175 | LPIN1 | -1.6878 | 1.65E-07 |
| 5293 | PIK3CD | -1.6429 | 1.67E-07 |
| 1282 | COL4A1 | -1.5916 | 1.72E-07 |
| 79651 | RHBDF2 | -1.6155 | 2.65E-07 |
| 58499 | ZNF462 | -1.6123 | 2.93E-07 |
| 3097 | HIVEP2 | -1.6197 | 3.23E-07 |
| 85377 | MICALL1 | -1.8873 | 3.57E-07 |
| 4478 | MSN | -1.5703 | 4.30E-07 |
| 9469 | CHST3 | -1.5861 | 4.32E-07 |
| 391 | RHOG | -1.5558 | 4.38E-07 |
| 50628 | GEMIN4 | -1.6093 | 4.92E-07 |
| 5465 | PPARA | -1.5703 | 5.34E-07 |
| 4162 | MCAM | -1.5066 | 5.40E-07 |
| 9466 | IL27RA | -1.5989 | 9.55E-07 |
| 23774 | BRD1 | -1.5072 | 9.56E-07 |
| 3689 | ITGB2 | -1.4986 | 9.56E-07 |
| 57567 | ZNF319 | -1.5199 | 9.72E-07 |
| 5361 | PLXNA1 | -1.556 | 1.00E-06 |
| 133522 | PPARGC1B | -1.5278 | 1.01E-06 |
| 27043 | PELP1 | -1.4987 | 1.01E-06 |
| 23046 | KIF21B | -1.5277 | 1.16E-06 |
| 7862 | BRPF1 | -1.5783 | 1.17E-06 |
| 9688 | N193 | -1.4751 | 1.20E-06 |
| 5351 | PLOD1 | -1.4949 | 1.21E-06 |
| 10507 | SEMA4D | -1.5127 | 1.24E-06 |
| 10525 | HYOU1 | -1.5507 | 1.25E-06 |
| 6497 | SKI | -1.453 | 1.49E-06 |
| 6541 | SLC7A1 | -1.4838 | 1.58E-06 |
| 7133 | TNFRSF1B | -1.4921 | 1.69E-06 |
| 257106 | ARHGAP30 | -1.4893 | 1.84E-06 |
| 25902 | MTHFD1L | -1.561 | 1.84E-06 |
| 83439 | TCF7L1 | -1.4864 | 1.86E-06 |
| 10957 | PNRC1 | -1.4707 | 2.97E-06 |
| 140947 | C5orf20 | -1.4636 | 3.06E-06 |
| 54440 | SASH3 | -1.457 | 3.49E-06 |
| 64780 | MICAL1 | -1.4475 | 3.59E-06 |
| 3609 | ILF3 | -1.4414 | 3.70E-06 |
| 22808 | MRAS | -1.4404 | 3.77E-06 |
| 26031 | OSBPL3 | -1.3994 | 3.78E-06 |
| 4318 | MMP9 | -1.4176 | 3.80E-06 |
| 64115 | C10orf54 | -1.4465 | 3.95E-06 |
| 27074 | LAMP3 | -1.4453 | 4.11E-06 |
| 23 | ABCF1 | -1.3949 | 4.47E-06 |
| 3142 | HLX | -1.4277 | 5.07E-06 |
| 23240 | KIAA0922 | -1.4228 | 5.12E-06 |
| 4651 | MYO10 | -1.4185 | 5.14E-06 |
| 3059 | HCLS1 | -1.4152 | 5.15E-06 |
| 10479 | SLC9A6 | -1.4591 | 5.90E-06 |
| 54784 | ALKBH4 | -1.4335 | 5.98E-06 |
| 58504 | ARHGAP22 | -1.3967 | 6.24E-06 |
| 84617 | TUBB6 | -1.3869 | 6.79E-06 |
| 4864 | NPC1 | -1.4455 | 8.00E-06 |
| 81553 | FAM49A | -1.413 | 9.46E-06 |
| 164284 | APCDD1L | -1.3793 | 1.07E-05 |
| 871 | SERPINH1 | -1.3127 | 1.07E-05 |
| 867 | CBL | -1.3516 | 1.23E-05 |
| 5650 | KLK7 | -1.3653 | 1.25E-05 |
| 8440 | NCK2 | -1.3674 | 1.26E-05 |
| 6397 | SEC14L1 | -1.4063 | 1.27E-05 |
| 10403 | NDC80 | -1.3887 | 1.29E-05 |
| 113 | ADCY7 | -1.367 | 1.30E-05 |
| 53335 | BCL11A | -1.3545 | 1.47E-05 |
| 57622 | LRFN1 | -1.482 | 1.56E-05 |
| 9513 | FXR2 | -1.3139 | 1.56E-05 |
| 1871 | E2F3 | -1.7111 | 1.57E-05 |
| 3930 | LBR | -1.3412 | 1.57E-05 |
| 9656 | MDC1 | -1.336 | 1.60E-05 |
| 5336 | PLCG2 | -1.343 | 1.82E-05 |
| 1612 | DAPK1 | -1.3706 | 1.98E-05 |
| 128077 | LIX1L | -1.3596 | 2.03E-05 |
| 3383 | ICAM1 | -1.3186 | 2.20E-05 |
| 11182 | SLC2A6 | -1.331 | 2.24E-05 |
| 29123 | ANKRD11 | -1.3037 | 2.27E-05 |
| 23406 | COTL1 | -1.3259 | 2.57E-05 |
| 9882 | TBC1D4 | -1.3261 | 2.59E-05 |
| 23082 | PPRC1 | -1.3131 | 2.61E-05 |
| 3613 | IMPA2 | -1.5092 | 2.87E-05 |
| 2296 | FOXC1 | -1.8596 | 2.99E-05 |
| 84959 | UBASH3B | -1.3946 | 3.00E-05 |
| 84898 | PLXDC2 | -1.3064 | 3.02E-05 |
| 23476 | BRD4 | -1.2961 | 3.03E-05 |
| 2303 | FOXC2 | -1.8215 | 3.20E-05 |
| 57673 | BEND3 | -1.2979 | 3.45E-05 |
| 2000 | ELF4 | -1.2673 | 3.68E-05 |
| 5954 | RCN1 | -1.3052 | 3.69E-05 |
| 910 | CD1B | -1.2939 | 3.70E-05 |
| 9095 | TBX19 | -1.4159 | 3.85E-05 |
| 3198 | HOXA1 | -1.2519 | 3.91E-05 |
| 124460 | SNX20 | -1.292 | 4.24E-05 |
| 8140 | SLC7A5 | -1.2739 | 4.60E-05 |
| 162 | AP1B1 | -1.3353 | 4.83E-05 |
| 976 | CD97 | -1.28 | 4.83E-05 |
| 8175 | SF3A2 | -1.2484 | 4.87E-05 |
| 145864 | HAPLN3 | -1.2894 | 5.38E-05 |
| 155038 | GIMAP8 | -1.276 | 5.71E-05 |
| 5578 | PRKCA | -1.2596 | 5.73E-05 |
| 10019 | SH2B3 | -1.2582 | 5.76E-05 |
| 25851 | TECPR1 | -1.2703 | 5.78E-05 |
| 1230 | CCR1 | -1.254 | 6.38E-05 |
| 84451 | KIAA1804 | -1.2086 | 6.86E-05 |
| 3162 | HMOX1 | -1.2302 | 6.98E-05 |
| 9047 | SH2D2A | -1.273 | 6.99E-05 |
| 4851 | NOTCH1 | -1.2519 | 7.06E-05 |
| 79174 | CRELD2 | -1.3081 | 7.07E-05 |
| 4288 | MKI67 | -1.2243 | 7.08E-05 |
| 3560 | IL2RB | -1.2269 | 7.89E-05 |
| 23500 | DAAM2 | -1.2316 | 7.90E-05 |
| 196410 | METTL7B | -1.3291 | 8.06E-05 |
| 83937 | RASSF4 | -1.2316 | 8.42E-05 |
| 4245 | MGAT1 | -1.2361 | 8.44E-05 |
| 10523 | CHERP | -1.2334 | 8.80E-05 |
| 8645 | KCNK5 | -1.3974 | 9.18E-05 |
| 2568 | GABRP | -1.2246 | 9.53E-05 |
| 695 | BTK | -1.2241 | 0.00010104 |
| 5606 | MAP2K3 | -1.2277 | 0.00010138 |
| 57210 | SLC45A4 | -1.2192 | 0.00010268 |
| 169714 | QSOX2 | -1.223 | 0.00010456 |
| 55143 | CDCA8 | -1.2273 | 0.00010631 |
| 5551 | PRF1 | -1.2194 | 0.0001115 |
| 118788 | PIK3AP1 | -1.2356 | 0.00011162 |
| 92806 | CENPBD1 | -1.1782 | 0.0001121 |
| 22934 | RPIA | -1.2082 | 0.00011684 |
| 9880 | ZBTB39 | -1.158 | 0.00011981 |
| 54815 | GATAD2A | -1.1952 | 0.0001208 |
| 5184 | PEPD | -1.3632 | 0.00012084 |
| 54478 | FAM64A | -1.2975 | 0.00012372 |
| 141 | ADPRH | -1.2037 | 0.00012573 |
| 10482 | NXF1 | -1.1815 | 0.00012691 |
| 55810 | FOXJ2 | -1.1854 | 0.00012708 |
| 134637 | ADAT2 | -1.218 | 0.00013034 |
| 55691 | FRMD4A | -1.2052 | 0.00013058 |
| 9711 | KIAA0226 | -1.1906 | 0.00013862 |
| 1522 | CTSZ | -1.1675 | 0.00014537 |
| 717 | C2 | -1.1781 | 0.0001463 |
| 2305 | FOXM1 | -1.1845 | 0.00015756 |
| 64359 | NXN | -1.1963 | 0.00016025 |
| 10288 | LILRB2 | -1.2062 | 0.00016693 |
| 1184 | CLCN5 | -1.1839 | 0.00017086 |
| 7297 | TYK2 | -1.1762 | 0.0001723 |
| 83706 | FERMT3 | -1.1875 | 0.0001728 |
| 81928 | CABLES2 | -1.1769 | 0.00017574 |
| 51704 | GPRC5B | -1.222 | 0.00017597 |
| 135293 | PM20D2 | -1.1969 | 0.00018142 |
| 79930 | DOK3 | -1.1805 | 0.00018436 |
| 25975 | EGFL6 | -1.2169 | 0.00018446 |
| 9797 | TATDN2 | -1.1943 | 0.00018477 |
| 200424 | TET3 | -1.1783 | 0.00018482 |
| 1051 | CEBPB | -1.1512 | 0.00018489 |
| 5026 | P2RX5 | -1.1899 | 0.00018509 |
| 27242 | TNFRSF21 | -1.1736 | 0.00019534 |
| 4001 | LMNB1 | -1.1468 | 0.00019682 |
| 115992 | RNF166 | -1.177 | 0.00019893 |
| 5727 | PTCH1 | -1.1723 | 0.00020221 |
| 5428 | POLG | -1.2568 | 0.00020901 |
| 4542 | MYO1F | -1.1713 | 0.00021231 |
| 79690 | GAL3ST4 | -1.1641 | 0.00021608 |
| 3071 | NCKAP1L | -1.1621 | 0.0002173 |
| 2113 | ETS1 | -1.1694 | 0.00021789 |
| 51172 | NAGPA | -1.1527 | 0.00021822 |
| 56833 | SLAMF8 | -1.1625 | 0.00022016 |
| 10763 | NES | -1.1613 | 0.00022511 |
| 90231 | KIAA2013 | -1.1456 | 0.00023169 |
| 80833 | APOL3 | -1.1265 | 0.00023172 |
| 4811 | NID1 | -1.1444 | 0.00023352 |
| 9791 | PTDSS1 | -1.2213 | 0.00023406 |
| 84706 | GPT2 | -1.1766 | 0.00023687 |
| 10749 | KIF1C | -1.1462 | 0.00024324 |
| 3061 | HCRTR1 | -1.1546 | 0.00024895 |
| 91010 | FMNL3 | -1.2483 | 0.00025046 |
| 7942 | TFEB | -1.153 | 0.00025122 |
| 7454 | WAS | -1.1547 | 0.00025501 |
| 171023 | ASXL1 | -1.1484 | 0.00025635 |
| 7298 | TYMS | -1.1073 | 0.00025769 |
| 5093 | PCBP1 | -1.114 | 0.00026223 |
| 2833 | CXCR3 | -1.1496 | 0.00026765 |
| 8570 | KHSRP | -1.1656 | 0.00027442 |
| 29986 | SLC39A2 | -1.1055 | 0.00027668 |
| 64333 | ARHGAP9 | -1.1435 | 0.00027996 |
| 6614 | SIGLEC1 | -1.1148 | 0.0002968 |
| 1054 | CEBPG | -1.3808 | 0.00029712 |
| 22821 | RASA3 | -1.1421 | 0.0002973 |
| 283897 | C16orf54 | -1.1396 | 0.00029754 |
| 57699 | CPNE5 | -1.1257 | 0.00030079 |
| 1994 | ELAVL1 | -1.1079 | 0.00031001 |
| 54910 | SEMA4C | -1.1066 | 0.00031225 |
| 4174 | MCM5 | -1.1329 | 0.00031679 |
| 197259 | MLKL | -1.1361 | 0.00032156 |
| 7124 | TNF | -1.1221 | 0.00032724 |
| 23381 | SMG5 | -1.0943 | 0.0003273 |
| 10318 | TNIP1 | -1.0967 | 0.00033067 |
| 23019 | CNOT1 | -1.1039 | 0.00034193 |
| 51442 | VGLL1 | -1.1247 | 0.00034338 |
| 5970 | RELA | -1.0959 | 0.0003439 |
| 51678 | MPP6 | -1.1105 | 0.000361 |
| 23616 | SH3BP1 | -1.0698 | 0.00036387 |
| 3570 | IL6R | -1.1255 | 0.00036663 |
| 5476 | CTSA | -1.1044 | 0.00037907 |
| 10943 | MSL3 | -1.1052 | 0.00039068 |
| 5160 | PDHA1 | -1.8886 | 0.00039278 |
| 7277 | TUBA4A | -1.0892 | 0.00040101 |
| 9770 | RASSF2 | -1.1187 | 0.00040245 |
| 6689 | SPIB | -1.0665 | 0.000418 |
| 6624 | FSCN1 | -1.1133 | 0.0004191 |
| 8291 | DYSF | -1.1471 | 0.00042074 |
| 1824 | DSC2 | -1.1152 | 0.0004344 |
| 54849 | DEF8 | -1.1287 | 0.00043462 |
| 3588 | IL10RB | -1.0978 | 0.00044066 |
| 898 | CCNE1 | -1.3862 | 0.00044163 |
| 6915 | TBXA2R | -1.1167 | 0.00045608 |
| 3787 | KCNS1 | -1.3875 | 0.00046406 |
| 29968 | PSAT1 | -1.102 | 0.00046475 |
| 55114 | ARHGAP17 | -1.1071 | 0.00046793 |
| 5598 | MAPK7 | -1.2732 | 0.00047158 |
| 1439 | CSF2RB | -1.0986 | 0.00047755 |
| 11186 | RASSF1 | -1.1031 | 0.00048106 |
| 4261 | CIITA | -1.1001 | 0.0004838 |
| 8514 | KCNAB2 | -1.1035 | 0.00048957 |
| 10939 | AFG3L2 | -1.0951 | 0.00049375 |
| 2534 | FYN | -1.1012 | 0.00049607 |
| 9700 | ESPL1 | -1.0721 | 0.00049754 |
| 3455 | IFNAR2 | -1.0881 | 0.00049795 |
| 864 | RUNX3 | -1.1024 | 0.0004983 |
| 6891 | TAP2 | -1.0813 | 0.00051535 |
| 8761 | PABPC4 | -1.0978 | 0.00052075 |
| 84230 | LRRC8C | -1.0941 | 0.00052279 |
| 7111 | TMOD1 | -1.2136 | 0.00052793 |
| 64926 | RASAL3 | -1.0821 | 0.00053467 |
| 29842 | TFCP2L1 | -1.1231 | 0.00054493 |
| 3783 | KCNN4 | -1.0945 | 0.00054788 |
| 2588 | GALNS | -1.0847 | 0.00054929 |
| 54 | ACP5 | -1.0709 | 0.00054945 |
| 23533 | PIK3R5 | -1.0915 | 0.00055407 |
| 9834 | KIAA0125 | -1.0838 | 0.00055552 |
| 4050 | LTB | -1.0874 | 0.00056054 |
| 4839 | NOP2 | -1.0945 | 0.00058245 |
| 2189 | FANCG | -1.0553 | 0.00058839 |
| 84079 | ANKRD27 | -1.0813 | 0.00060126 |
| 9922 | IQSEC1 | -1.0826 | 0.00061629 |
| 64764 | CREB3L2 | -1.0824 | 0.00064007 |
| 285598 | ARL10 | -1.085 | 0.00065101 |
| 85453 | TSPYL5 | -1.2188 | 0.00065326 |
| 7453 | WARS | -1.0355 | 0.00067596 |
| 81624 | DIAPH3 | -1.0551 | 0.00068247 |
| 790 | CAD | -1.0495 | 0.00069255 |
| 54438 | GFOD1 | -1.0506 | 0.00070493 |
| 6838 | SURF6 | -1.0724 | 0.00070968 |
| 23201 | FAM168A | -1.0674 | 0.00072151 |
| 100 | ADA | -1.059 | 0.0007303 |
| 79192 | IRX1 | -1.0627 | 0.00074857 |
| 10052 | GJC1 | -1.0489 | 0.00076604 |
| 3683 | ITGAL | -1.0681 | 0.00076666 |
| 9943 | OXSR1 | -1.0466 | 0.00076883 |
| 4600 | MX2 | -1.0627 | 0.00077511 |
| 5420 | PODXL | -1.0384 | 0.00077768 |
| 54733 | SLC35F2 | -1.0544 | 0.00078342 |
| 440400 | RNASEK | -1.0542 | 0.00079217 |
| 2091 | FBL | -1.053 | 0.00079276 |
| 5754 | PTK7 | -1.0657 | 0.00079413 |
| 8728 | ADAM19 | -1.0591 | 0.00083801 |
| 22955 | SCMH1 | -1.0654 | 0.00084104 |
| 1200 | TPP1 | -1.0291 | 0.00084319 |
| 7456 | WIPF1 | -1.062 | 0.00084364 |
| 9046 | DOK2 | -1.0531 | 0.00084891 |
| 547 | KIF1A | -1.0585 | 0.00085644 |
| 55704 | CCDC88A | -1.048 | 0.0008624 |
| 6875 | TAF4B | -1.0375 | 0.0008671 |
| 55154 | MSTO1 | -1.0625 | 0.0008679 |
| 3482 | IGF2R | -1.0225 | 0.00087188 |
| 55506 | H2AFY2 | -1.0561 | 0.000877 |
| 23076 | RRP1B | -1.0548 | 0.00089226 |
| 79156 | PLEKHF1 | -1.0489 | 0.00089299 |
| 24149 | ZNF318 | -1.051 | 0.00094849 |
| 221545 | C6orf136 | -1.0509 | 0.00094977 |
| 578 | BAK1 | -1.0362 | 0.00095418 |
| 2592 | GALT | -1.0478 | 0.00096743 |
| 27240 | SIT1 | -1.0184 | 0.00097015 |
| 272 | AMPD3 | -1.0475 | 0.0009885 |
| 145567 | TTC7B | -1.013 | 0.00099136 |
| 23164 | MPRIP | -1.0485 | 0.00099384 |
| 811 | CALR | -1.042 | 0.001002 |
| 23562 | CLDN14 | -1.0283 | 0.0010033 |
| 53827 | FXYD5 | -1.0012 | 0.0010049 |
| 23648 | SSBP3 | -1.0303 | 0.0010191 |
| 7145 | TNS1 | -1.0007 | 0.0010456 |
| 54842 | MFSD6 | -3.1612 | 0.0010627 |
| 1794 | DOCK2 | -1.0396 | 0.0010674 |
| 6868 | ADAM17 | -1.0377 | 0.0010812 |
| 25771 | TBC1D22A | -1.029 | 0.0011256 |
| 187 | APLNR | -1.0112 | 0.0011524 |
| 140901 | STK35 | -1.0295 | 0.0011619 |
| 84467 | FBN3 | -1.0206 | 0.0011622 |
| 26227 | PHGDH | -1.033 | 0.0011738 |
| 147138 | TMC8 | -1.0249 | 0.0011754 |
| 152007 | GLIPR2 | -1.0191 | 0.0011837 |
| 3759 | KCNJ2 | -1.0014 | 0.0012124 |
| 9990 | SLC12A6 | -1.0267 | 0.0012169 |
| 5653 | KLK6 | -1.0314 | 0.0012287 |
| 10650 | SLMO1 | -1.0252 | 0.0012857 |
| 375035 | SFT2D2 | -1.0015 | 0.0013323 |
| 79172 | CENPO | -1.0133 | 0.001347 |
| 5830 | PEX5 | -1.0194 | 0.0014316 |
| 3936 | LCP1 | -1.0167 | 0.0014805 |
| 51729 | WBP11 | -1.2649 | 0.0015436 |
| 9792 | SERTAD2 | -1.0115 | 0.0015457 |
| 55646 | LYAR | -1.0829 | 0.0015477 |
| 10461 | MERTK | -1.0066 | 0.0016084 |
| 81704 | DOCK8 | -1.0073 | 0.0016333 |
| 79080 | CCDC86 | -1.1069 | 0.0016436 |
| 170575 | GIMAP1 | -1.0062 | 0.0016581 |
| 89845 | ABCC10 | -1.0022 | 0.0016901 |
| 9889 | ZBED4 | -1.0069 | 0.001721 |
| 254428 | SLC41A1 | -1.0086 | 0.0017361 |
| 84306 | PDCD2L | -1.5656 | 0.0017641 |
| 8934 | RAB7L1 | -1.002 | 0.00179 |
| 1299 | COL9A3 | -4.2415 | 0.001872 |
| 57180 | ACTR3B | -1.0078 | 0.0019328 |
| 120071 | GYLTL1B | -1.2272 | 0.0024853 |
| 54331 | GNG2 | -2.6582 | 0.0029338 |
| 705 | BYSL | -1.0284 | 0.0032179 |
| 55635 | DEPDC1 | -1.1636 | 0.0032689 |
| 7327 | UBE2G2 | -1.0695 | 0.0036122 |
| 3595 | IL12RB2 | -1.5186 | 0.004283 |
| 23165 | N1205 | -1.0496 | 0.0047601 |
| 6801 | STRN | -1.0238 | 0.005733 |
| 23029 | RBM34 | -1.0288 | 0.0061215 |
| 10526 | IPO8 | -1.0133 | 0.0065502 |
| 84908 | FAM136A | -1.3707 | 0.007572 |
| 144568 | A2ML1 | -3.0621 | 0.0089911 |
| 23590 | PDSS1 | -1.029 | 0.0098361 |

1. Down-regulated genes

| **Gene ID** | **Gene Symbol** | **combined ES** | **combined p-value(fdr)** |
| --- | --- | --- | --- |
| 134147 | CMBL | 1.9233 | 4.29E-09 |
| 1843 | DUSP1 | 1.7381 | 2.17E-08 |
| 153562 | MARVELD2 | 1.7058 | 8.99E-08 |
| 26996 | GPR160 | 1.8247 | 1.06E-07 |
| 3169 | FOXA1 | 2.3038 | 1.67E-07 |
| 57669 | EPB41L5 | 1.6701 | 2.26E-07 |
| 158158 | RASEF | 1.6221 | 2.62E-07 |
| 27089 | UQCRQ | 1.5901 | 3.71E-07 |
| 150678 | MYEOV2 | 1.6048 | 4.06E-07 |
| 79083 | MLPH | 11.993 | 5.48E-07 |
| 79875 | THSD4 | 2.1243 | 8.41E-07 |
| 150590 | C2orf15 | 1.5304 | 8.98E-07 |
| 10551 | AGR2 | 1.6211 | 1.25E-06 |
| 91074 | ANKRD30A | 1.5391 | 1.30E-06 |
| 79858 | NEK11 | 1.4965 | 1.35E-06 |
| 148327 | CREB3L4 | 1.4464 | 4.39E-06 |
| 64800 | EFCAB6 | 1.4295 | 4.82E-06 |
| 585 | BBS4 | 1.877 | 4.91E-06 |
| 7033 | TFF3 | 1.6601 | 6.28E-06 |
| 4437 | MSH3 | 1.3985 | 7.72E-06 |
| 7494 | XBP1 | 1.6796 | 8.39E-06 |
| 55614 | KIF16B | 1.3968 | 9.22E-06 |
| 57221 | KIAA1244 | 1.3467 | 1.33E-05 |
| 5205 | ATP8B1 | 1.3735 | 1.38E-05 |
| 64792 | RABL5 | 1.3548 | 2.07E-05 |
| 79818 | ZNF552 | 1.3592 | 2.19E-05 |
| 155465 | AGR3 | 1.3332 | 3.02E-05 |
| 403 | ARL3 | 1.3514 | 3.06E-05 |
| 2947 | GSTM3 | 1.3106 | 3.09E-05 |
| 771 | CA12 | 1.3228 | 3.15E-05 |
| 27324 | TOX3 | 1.3975 | 3.21E-05 |
| 170685 | NUDT10 | 2.4717 | 3.38E-05 |
| 2099 | ESR1 | 1.7342 | 3.46E-05 |
| 163720 | CYP4Z2P | 1.5589 | 3.51E-05 |
| 84953 | MICALCL | 1.3131 | 3.70E-05 |
| 644 | BLVRA | 1.2783 | 4.60E-05 |
| 222171 | PRR15 | 1.2881 | 4.84E-05 |
| 55103 | RALGPS2 | 1.2687 | 5.08E-05 |
| 202915 | TMEM184A | 8.8156 | 5.40E-05 |
| 54970 | TTC12 | 1.2686 | 5.40E-05 |
| 65055 | REEP1 | 1.7209 | 5.42E-05 |
| 8382 | NME5 | 1.3037 | 5.55E-05 |
| 6500 | SKP1 | 1.2617 | 5.76E-05 |
| 25800 | SLC39A6 | 1.2672 | 6.16E-05 |
| 121441 | NEDD1 | 3.6729 | 6.59E-05 |
| 23171 | GPD1L | 1.4356 | 6.60E-05 |
| 253959 | RALGAPA1 | 1.2515 | 7.06E-05 |
| 84708 | LNX1 | 1.2558 | 7.06E-05 |
| 9140 | ATG12 | 1.245 | 7.49E-05 |
| 124152 | IQCK | 1.2497 | 7.90E-05 |
| 55105 | GPATCH2 | 1.2372 | 8.64E-05 |
| 2353 | FOS | 1.3449 | 8.92E-05 |
| 51340 | CRNKL1 | 1.2276 | 9.18E-05 |
| 112479 | ERI2 | 1.2289 | 9.19E-05 |
| 214 | ALCAM | 1.2348 | 9.40E-05 |
| 96459 | FNIP1 | 1.2188 | 9.67E-05 |
| 149473 | CCDC24 | 1.2363 | 0.00010457 |
| 123016 | TTC8 | 1.2246 | 0.00011653 |
| 158293 | FAM120AOS | 1.5843 | 0.0001167 |
| 114327 | EFHC1 | 1.2185 | 0.0001182 |
| 2625 | GATA3 | 1.3067 | 0.0001182 |
| 10916 | MAGED2 | 1.3218 | 0.00012576 |
| 27236 | ARFIP1 | 1.1999 | 0.00012728 |
| 8821 | INPP4B | 1.4953 | 0.00013068 |
| 51265 | CDKL3 | 1.2049 | 0.00013119 |
| 51760 | SYT17 | 1.2904 | 0.00013128 |
| 11257 | TP53TG1 | 1.2037 | 0.0001325 |
| 79846 | C7orf63 | 1.2104 | 0.00013863 |
| 6337 | SCNN1A | 1.2581 | 0.00014118 |
| 25803 | SPDEF | 1.2051 | 0.00014712 |
| 55837 | EAPP | 1.1973 | 0.0001507 |
| 25823 | TPSG1 | 1.1581 | 0.00015642 |
| 80021 | TMEM62 | 1.204 | 0.00016296 |
| 1040 | CDS1 | 1.1896 | 0.00016726 |
| 54940 | OCIAD1 | 1.1766 | 0.0001841 |
| 92291 | CAPN13 | 1.2408 | 0.00018425 |
| 10229 | COQ7 | 1.1826 | 0.00018436 |
| 10512 | SEMA3C | 1.1979 | 0.00018468 |
| 55973 | BCAP29 | 1.1839 | 0.00018484 |
| 55805 | LRP2BP | 1.3416 | 0.00018526 |
| 92104 | TTC30A | 1.17 | 0.00018849 |
| 25976 | TIPARP | 1.1791 | 0.00019194 |
| 8766 | RAB11A | 1.1413 | 0.00019563 |
| 1528 | CYB5A | 1.2361 | 0.00019862 |
| 9522 | SCAMP1 | 1.1736 | 0.00020271 |
| 1846 | DUSP4 | 1.3936 | 0.00020377 |
| 8100 | IFT88 | 1.1653 | 0.00020757 |
| 79170 | PRR15L | 1.2119 | 0.00021308 |
| 27075 | TSPAN13 | 1.2741 | 0.0002135 |
| 51397 | COMMD10 | 1.1684 | 0.00021785 |
| 252884 | ZNF396 | 1.1642 | 0.00021896 |
| 120224 | TMEM45B | 1.3795 | 0.00022111 |
| 55930 | MYO5C | 1.139 | 0.00023387 |
| 406991 | MIR21 | 1.1609 | 0.0002363 |
| 89894 | TMEM116 | 1.1573 | 0.0002433 |
| 195814 | SDR16C5 | 1.5714 | 0.00024335 |
| 84274 | COQ5 | 1.1578 | 0.00024809 |
| 51523 | CXXC5 | 1.2487 | 0.00024988 |
| 9135 | RABEP1 | 1.1573 | 0.00025005 |
| 6505 | SLC1A1 | 2.1148 | 0.00025537 |
| 5494 | PPM1A | 1.1328 | 0.00027091 |
| 91893 | FDXACB1 | 1.1508 | 0.00027122 |
| 57415 | C3orf14 | 1.1085 | 0.00028177 |
| 7358 | UGDH | 1.1505 | 0.00029689 |
| 23002 | DAAM1 | 1.1423 | 0.00030278 |
| 255520 | ELMOD2 | 1.1366 | 0.00030533 |
| 5167 | ENPP1 | 1.1415 | 0.00031528 |
| 400451 | FAM174B | 1.1416 | 0.00031666 |
| 7031 | TFF1 | 1.2154 | 0.00031712 |
| 54014 | BRWD1 | 1.1369 | 0.00031793 |
| 90355 | C5orf30 | 1.1534 | 0.00032766 |
| 4695 | NDUFA2 | 1.1208 | 0.00033935 |
| 199974 | CYP4Z1 | 1.332 | 0.00035235 |
| 51097 | SCCPDH | 1.1558 | 0.00035251 |
| 127700 | OSCP1 | 1.1185 | 0.00036334 |
| 55793 | FAM63A | 1.1543 | 0.00036478 |
| 10142 | AKAP9 | 1.1111 | 0.00036568 |
| 160418 | TMTC3 | 1.1254 | 0.00036631 |
| 126823 | KLHDC9 | 1.1253 | 0.00036798 |
| 401546 | C9orf152 | 1.1198 | 0.00037879 |
| 222484 | LNX2 | 1.1229 | 0.00038029 |
| 51133 | KCTD3 | 1.1018 | 0.00038058 |
| 4602 | MYB | 1.1059 | 0.00038113 |
| 5229 | PGGT1B | 1.1259 | 0.00039823 |
| 4832 | NME3 | 1.0968 | 0.0004069 |
| 158584 | FAAH2 | 1.246 | 0.00041005 |
| 6165 | RPL35A | 1.0919 | 0.0004196 |
| 5204 | PFDN5 | 1.1096 | 0.00042141 |
| 122970 | ACOT4 | 1.2662 | 0.0004283 |
| 51643 | TMBIM4 | 1.2867 | 0.00044071 |
| 80267 | EDEM3 | 3.9278 | 0.00045056 |
| 132949 | AASDH | 1.1043 | 0.00048057 |
| 54536 | EXOC6 | 1.0994 | 0.00051461 |
| 4646 | MYO6 | 1.1605 | 0.00052148 |
| 51495 | PTPLAD1 | 1.0993 | 0.00052625 |
| 202018 | TAPT1 | 1.1973 | 0.00053973 |
| 130888 | FBXO36 | 1.0901 | 0.00055314 |
| 5304 | PIP | 1.1 | 0.00055399 |
| 1452 | CSNK1A1 | 1.0781 | 0.00055419 |
| 9 | NAT1 | 1.0802 | 0.00055897 |
| 79667 | FLJ13197 | 1.054 | 0.00056994 |
| 55259 | CASC1 | 1.0782 | 0.00059148 |
| 84059 | GPR98 | 1.0752 | 0.00063117 |
| 10254 | STAM2 | 1.08 | 0.00065543 |
| 51361 | HOOK1 | 1.0756 | 0.00067679 |
| 91147 | TMEM67 | 1.0597 | 0.00068811 |
| 79659 | DYNC2H1 | 1.1426 | 0.00071807 |
| 28981 | IFT81 | 1.0655 | 0.00072563 |
| 219402 | MTIF3 | 1.0488 | 0.00076165 |
| 80736 | SLC44A4 | 1.0434 | 0.00077566 |
| 7162 | TPBG | 1.0574 | 0.00079326 |
| 84914 | ZNF587 | 1.0659 | 0.00079828 |
| 5269 | SERPINB6 | 1.0544 | 0.0008103 |
| 56929 | FEM1C | 1.0587 | 0.00081682 |
| 8870 | IER3 | 1.0623 | 0.00082413 |
| 53340 | SPA17 | 1.0504 | 0.00084998 |
| 23158 | TBC1D9 | 1.0571 | 0.00085105 |
| 150274 | HSCB | 1.0555 | 0.00087365 |
| 56521 | DNAJC12 | 1.0542 | 0.00089193 |
| 133015 | PACRGL | 1.0813 | 0.00091404 |
| 127733 | UBXN10 | 1.0529 | 0.00097037 |
| 55112 | WDR60 | 1.0467 | 0.00098142 |
| 10040 | TOM1L1 | 1.049 | 0.00098236 |
| 64087 | MCCC2 | 1.0343 | 0.00099861 |
| 11103 | KRR1 | 1.0435 | 0.0010022 |
| 22869 | ZNF510 | 1.1274 | 0.0010462 |
| 81563 | C1orf21 | 1.0504 | 0.0010483 |
| 64284 | RAB17 | 1.0428 | 0.001052 |
| 51141 | INSIG2 | 1.0236 | 0.0011155 |
| 9649 | RALGPS1 | 1.0885 | 0.0011243 |
| 57149 | LYRM1 | 1.0396 | 0.0011344 |
| 23047 | PDS5B | 1.0149 | 0.0011419 |
| 23443 | SLC35A3 | 1.0324 | 0.0011432 |
| 5618 | PRLR | 1.0172 | 0.0011432 |
| 25837 | RAB26 | 1.0269 | 0.0011747 |
| 9488 | PIGB | 1.0209 | 0.0011805 |
| 51808 | PHAX | 1.0322 | 0.0011834 |
| 901 | CCNG2 | 1.0312 | 0.0011849 |
| 81579 | PLA2G12A | 1.0338 | 0.0011863 |
| 80184 | CEP290 | 1.0324 | 0.0012011 |
| 90693 | CCDC126 | 1.0161 | 0.0012045 |
| 286451 | YIPF6 | 1.0329 | 0.0012217 |
| 29063 | ZCCHC4 | 1.024 | 0.0012371 |
| 79962 | DNAJC22 | 1.1574 | 0.0012462 |
| 54502 | RBM47 | 1.0262 | 0.0012722 |
| 10234 | LRRC17 | 1.0149 | 0.0013325 |
| 1360 | CPB1 | 1.0733 | 0.0014512 |
| 521 | ATP5I | 1.0139 | 0.0014581 |
| 56674 | TMEM9B | 1.0234 | 0.0015107 |
| 90362 | FAM110B | 1.0087 | 0.0015839 |
| 5825 | ABCD3 | 1.0101 | 0.0016331 |
| 54677 | CROT | 1.0071 | 0.0016717 |
| 2967 | GTF2H3 | 1.0014 | 0.001759 |
| 2922 | GRP | 1.0062 | 0.0018216 |
| 54843 | SYTL2 | 1.0672 | 0.0018389 |
| 118491 | TTC18 | 1.0044 | 0.0018857 |
| 51004 | COQ6 | 1.0406 | 0.0019091 |
| 323 | APBB2 | 1.0688 | 0.00193 |
| 57182 | ANKRD50 | 1.0024 | 0.0021228 |
| 9748 | SLK | 1.8824 | 0.0025265 |
| 114932 | MRFAP1L1 | 1.0306 | 0.0025315 |
| 652 | BMP4 | 1.0048 | 0.0026656 |
| 5926 | ARID4A | 1.2116 | 0.0029712 |
| 138804 | OR13C4 | 3.9734 | 0.0031536 |
| 85014 | TMEM141 | 1.0237 | 0.0034933 |
| 55333 | SYNJ2BP | 1.0835 | 0.0037274 |
| 79979 | TRMT2B | 1.0416 | 0.0037958 |
| 220388 | CCDC89 | 1.0216 | 0.0049054 |
| 3955 | LFNG | 1.3326 | 0.0053466 |
| 889 | KRIT1 | 1.4261 | 0.0071982 |
| 5364 | PLXNB1 | 1.0091 | 0.0080683 |
| 79760 | GEMIN7 | 1.4109 | 0.0097767 |
