# Supplementary material for: Molecular Features of Triple Negative Breast Cancer: Microarray Evidence and Further Integrated Analysis
Source: PLoS One. 2015 Jun 23;10(6):e0129842. doi: 10.1371/journal.pone.0129842 (PMC4478040; doi:10.1371/journal.pone.0129842)
Supplement: S3 Table — (DOC) [file pone.0129842.s003.doc]

**S3 Table. The annotation of edges connecting the top 10 up- and down-regulated DEGs directly or indirectly**

| **Edge ID** | **Node 1** | **Node2** | **Name** | **Detection Method** | **Detection Method ID** | **Interaction** | **Interaction Type** | **Author** | **PMID** |
| --- | --- | --- | --- | --- | --- | --- | --- | --- | --- |
| 1 | RP11-297A16.3 | MYEOV2 | 8453 (688167) 150678 | affinity chromatography technology | MI:0004 | 688167 | physical association | Bennett EJ (2010) | 21145461 |
| 2 | RP11-297A16.3 | CMBL | 8453 (243445) 134147 | affinity chromatography technology | MI:0004 | 243445 | physical association | Ewing RM (2007) | 17353931 |
| 3 | RP11-297A16.3 | MLPH | 8453 (688162) 79083 | affinity chromatography technology | MI:0004 | 688162 | physical association | Bennett EJ (2010) | 21145461 |
| 4 | HMG20 | CMBL | 7316 (847822) 134147 | affinity chromatography technology | MI:0004 | 847822 | physical association | Emanuele MJ (2011) | 21963094 |
| 5 | BIS | CMBL | 9531 (876294) 134147 | affinity chromatography technology | MI:0004 | 876294 | physical association | Chen Y (2013) | 23824909 |
| 6 | BIS | BMF | 9531 (876276) 90427 | affinity chromatography technology | MI:0004 | 876276 | physical association | Chen Y (2013) | 23824909 |
| 7 | BMF | GS1 | 90427 (286119) 4644 | pull down | MI:0096 | 286119 | direct interaction | Puthalakath H (2001) | 11546872 |
| 8 | GS1 | MLPH | 4644 (301010) 79083 | pull down | MI:0096 | 301010 | direct interaction | Nagashima K (2002) | 12062444 |
| 9 | MLPH | GS1 | 79083 (252018) 4644 | affinity chromatography technology | MI:0004 | 252018 | physical association | Passeron T (2004) | 15059972 |
| 10 | JNK | BMF | 5599 (816439) 90427 | enzymatic study | MI:0415 | 816439 | direct interaction | Lei K (2003) | 12591950 |
| 11 | JNK | DUSP1 | 5599 (303628) 1843 | affinity chromatography technology | MI:0004 | 303628 | physical association | Slack DN (2001) | 11278799 |
| 12 | DUSP1 | JNK | 1843 (303625) 5599 | two hybrid | MI:0018 | 303625 | direct interaction | Slack DN (2001) | 11278799 |
| 13 | DUSP1 | HMG20 | 1843 (684441) 7316 | affinity chromatography technology | MI:0004 | 684441 | physical association | Xie P (2009) | 19117950 |
| 14 | HMG20 | DUSP1 | 7316 (609624) 1843 | affinity chromatography technology | MI:0004 | 609624 | physical association | Lee KA (2011) | 21987572 |
| 15 | HMG20 | EPB41L5 | 7316 (621055) 57669 | affinity chromatography technology | MI:0004 | 621055 | physical association | Danielsen JM (2011) | 21139048 |
| 16 | HMG20 | SNX8 | 7316 (618411) 29886 | affinity chromatography technology | MI:0004 | 618411 | physical association | Danielsen JM (2011) | 21139048 |
| 17 | Dlgh4 | SNX8 | 13385 (830232) 29886 | unspecified method | MI:0686 | 830232 | direct interaction | Arbuckle MI (2010) | 20467438 |
| 18 | Dlgh4 | BCL9L | 13385 (830238) 283149 | unspecified method | MI:0686 | 830238 | direct interaction | Arbuckle MI (2010) | 20467438 |
| 19 | NRF1 | SNX8 | 4899 (917204) 29886 | imaging technique | MI:0428 | 917204 | colocalization | Satoh J (2013) | 24250222 |
| 20 | HMG20 | KLF13 | 7316 (628185) 51621 | affinity chromatography technology | MI:0004 | 628185 | physical association | Kim W (2011) | 21906983 |
| 21 | NRF1 | KLF13 | 4899 (916669) 51621 | imaging technique | MI:0428 | 916669 | colocalization | Satoh J (2013) | 24250222 |
| 22 | HMG20 | UQCRQ | 7316 (625446) 27089 | affinity chromatography technology | MI:0004 | 625446 | physical association | Wagner SA (2011) | 21890473 |
| 23 | NRF1 | AKIRIN2 | 4899 (916308) 55122 | imaging technique | MI:0428 | 916308 | colocalization | Satoh J (2013) | 24250222 |
| 24 | HMG20 | AKIRIN2 | 7316 (622601) 55122 | affinity chromatography technology | MI:0004 | 622601 | physical association | Danielsen JM (2011) | 21139048 |
| 25 | Hua | AKIRIN2 | 1994 (662862) 55122 | affinity chromatography technology | MI:0004 | 662862 | physical association | Abdelmohsen K (2009) | 19322201 |
| 26 | IL17RA | HMG20 | 23765 (341213) 7316 | affinity chromatography technology | MI:0004 | 341213 | physical association | Rong Z (2007) | 17346928 |
| 27 | HMG20 | MARVELD2 | 7316 (623556) 153562 | affinity chromatography technology | MI:0004 | 623556 | physical association | Wagner SA (2011) | 21890473 |
| 28 | HMG20 | KLHL36 | 7316 (629047) 79786 | affinity chromatography technology | MI:0004 | 629047 | physical association | Kim W (2011) | 21906983 |
| 29 | HMG20 | RASEF | 7316 (622411) 158158 | affinity chromatography technology | MI:0004 | 622411 | physical association | Danielsen JM (2011) | 21139048 |
| 30 | HMG20 | MLPH | 7316 (631895) 79083 | affinity chromatography technology | MI:0004 | 631895 | physical association | Kim W (2011) | 21906983 |
| 31 | COPS5 | KLHL36 | 10987 (689597) 79786 | affinity chromatography technology | MI:0004 | 689597 | physical association | Bennett EJ (2010) | 21145461 |
| 32 | CUL3 | KLHL36 | 8452 (686106) 79786 | affinity chromatography technology | MI:0004 | 686106 | physical association | Bennett EJ (2010) | 21145461 |
| 33 | MYEOV2 | COPS5 | 150678 (874373) 10987 | affinity chromatography technology | MI:0004 | 874373 | physical association | Ebina M (2013) | 23776465 |
| 34 | COPS5 | MYEOV2 | 10987 (689659) 150678 | affinity chromatography technology | MI:0004 | 689659 | physical association | Bennett EJ (2010) | 21145461 |
| 35 | MYEOV2 | CUL3 | 150678 (874375) 8452 | affinity chromatography technology | MI:0004 | 874375 | physical association | Ebina M (2013) | 23776465 |
| 36 | CUL3 | MYEOV2 | 8452 (686595) 150678 | affinity chromatography technology | MI:0004 | 686595 | physical association | Bennett EJ (2010) | 21145461 |
| 37 | Hua | FOXA1 | 1994 (663630) 3169 | affinity chromatography technology | MI:0004 | 663630 | physical association | Abdelmohsen K (2009) | 19322201 |
| 38 | Hua | MARVELD2 | 1994 (662723) 153562 | affinity chromatography technology | MI:0004 | 662723 | physical association | Abdelmohsen K (2009) | 19322201 |
